# Waste No Time

**Published:** 1889-03

**Authors:** 


### Waste No Time.

After allowing yourself proper time for rest, don't live a single hour of your life without doing exactly what is to be done in that hour, and going straight through with it from beginning to end. Work, play, study, rest--whatever it is--take hold at once and finish it up squarly and clearly; then to the next thing without letting any moments drop out between. It is wonderful to see how many hours these prompt people make out of a day; it is as if they pick up the moments that so many of us lose. And if ever you find yourself where you have so many things pressing upon you that you hardly know how to begin, let us tell you a secret. *Take hold of the very first one that comes to hand*, in real earnest, and you will soon find that the rest will all fall into file, and follow after like a company of well-drilled soldiers; and though work may be hard to meet when it charges in a squad, it is easily vanquished if you can once bring it into line.--*Ex.*

